# Controlled Deposition of Single-Walled Carbon Nanotubes Doped Nanofibers Mats for Improving the Interlaminar Properties of Glass Fiber Hybrid Composites

**DOI:** 10.3390/polym15040957

**Published:** 2023-02-15

**Authors:** Arif Muhammad, Mkhululi Ncube, Nithish Aravinth, Jacob Muthu

**Affiliations:** 1 Faculty of Engineering & Technology, International Islamic University, Islamabad 44000, Pakistan; 2 Faculty of Engineering and the Built Environment, University of the Witwatersrand, Johannesburg 2000, South Africa; 3 Faculty of Engineering & Applied Science, University of Regina, Regina, SK S4S 0A2, Canada

**Keywords:** hybrid composites, aligned nanomats, functionalized SWCNTs, electrospinning, interlaminar region

## Abstract

The properties of glass fiber composites were improved by strengthening the interlaminar regions using electrospun nanofibers mats. However, the chaotic nature of the electrospinning process at the collector restricts the controlled deposition and alignment of nanofibers and limits the use of electrospun nanofibers as secondary reinforcements. Hence, auxiliary vertical electrodes were used, which drastically reduced the diameter of the nanofibers from 450 nm to 150 nm and also improved the alignment of nanofibers. The aligned nanofibers were then used for doping the functionalized single-walled carbon nanotubes (f-SWCNTs) with nanofibers, which controlled the inherent issues associated with SWCNTs such as agglomeration, poor dispersion, and alignment. This process produced f-SWCNTs doped nanofiber mats. A series of tensile, three-point flexural, and Charpy impact tests showed that 30 vol% glass fiber composites reinforced with 0.5 wt% of randomly oriented nanofiber (RONFs) mats improved the properties of the hybrid composites compared to 0.1 wt%, 0.2 wt%, and 1 wt% RONFs mats reinforced glass fiber hybrid composites. The increase in properties for 0.5 wt% composites was attributed to the higher specific surface area and resistance to the relative slip within the interlaminar regions. The 0.5 wt% RONFs were then used to produce 0.5 wt% of continuous-aligned nanofiber (CANFs) mats, which showed improved mechanical properties compared to 0.5 wt% randomly oriented nanofiber (RONFs) mats reinforced hybrid composites. The CANFs mats with reduced diameter increased the tensile strength, flexural strength, and impact resistance by 4.71%, 17.19%, and 20.53%, respectively, as compared to the random nanofiber mats. The increase in properties could be attributed to the reduced diameter, controlled deposition, and alignment of the nanofibers. Further, the highest increase in mechanical properties was achieved by the addition of f-SWCNTs doped CANFs mats strengthened hybrid composites, and the increase was 30.34% for tensile strength, 30.18% for flexural strength, and 132.29% for impact resistance, respectively. This improvement in properties was made possible by orderly alignments of f-SWCNTs within the nanofibers. The SEM images further confirmed that auxiliary vertical electrodes (AVEs) reduced the diameter, improved the alignment and molecular orientation of the nanofibers, and thus helped to reinforce the f-SWCNTs within the nanomats, which improved the properties of the glass hybrid composites.

## 1. Introduction

Glass fiber composites have a major role in various engineering applications due to their design flexibility, ease of manufacturing, and low weight compared to conventional materials such as steel [1,2]. However, the failure at the matrix-rich interlaminar regions due to poor mechanical properties is a cause of concern, and even with the higher volume fraction of glass fibers (60%), these matrix-rich regions exist [3]. Therefore, researchers are focused on improving the interlaminar regions by adding secondary reinforcements such as nanofillers [4,5,6], and among them, polymeric nanofibers are gaining greater importance due to their ease of manufacturing with unique mechanical properties [6].

Research works [7,8] show that continuous nanofibers have a higher molecular orientation with a better degree of crystallinity and, thus, they improve the properties of the interlaminar regions. Wang et al. [9] cited that continuous nanofibers could improve the mechanical properties along a particular direction and promote load-bearing capabilities. These results expounded the innovative ideas in manufacturing aligned and continuous nanofibers in the form of nanomats. These nanomats could develop a bridging mechanism within the interlaminar regions and be suitable interlaminar reinforcements for glass fiber hybrid composites. The continuous-aligned nanofibers (CANFs) mats will be thin, light in weight, and will not increase the weight of final composites. Moreover, they can reduce the length scale mismatch between the macroscale fibers and the polymer matrix molecular chains. In addition, these nanomats could be collected directly over the fiber mats and thus reduce the processing time/cost.

Several studies focus on using various nanoparticles prepared as mats for reinforcing composites such as graphene-embedded paper for electromagnetic interference shielding (EMI) [10],graphene/SiCnw nanostrctured films for improving the mechanical and thermal properties of carbon fiber composites [11]. Among them, the single-walled carbon nanotubes (SWCNTs), though they are comparatively expensive, have attracted researchers due to their exceptional mechanical properties with higher specific surface area [12]. However, the inherent issues, such as agglomeration, alignment, and hydrophobic nature, limit their potential as secondary reinforcements, which necessitated the innovative research ideas for using SWCNTs as interlaminar reinforcements [13]. One such direction could be to develop continuous-aligned nanofibers mats doped with SWCNTs.

Several techniques are being used for producing polymeric nanofibers, and among them, electrospinning is considered simple and versatile. However, the polymeric jets formed during electrospinning have two distinct phases: near field, and far field [14]. In the near field, the jet takes a straight path, and in the far field, it starts whipping into a complex path due to electrical, gravitational, and rheological forces. Though the far field yields nanosized fibers [15], the whipping leads to randomly oriented nanofibers, which defeats the objective of producing continuous and aligned nanofibers. Moreover, the nanosized fibers are vital for aligning and dispersing SWCNTs into the nanofibers mats. To overcome the whipping instability, researchers have used thin static collectors [16] or high-speed rotating collectors [17]. However, based on our experience, a wide rotating drum collector with auxiliary vertical electrodes (AVEs) seems to be the most suitable solution for controlling the whipping effect and also for improving the alignment of nanofibers within the nanomats. Moreover, AVEs have better control over the whipping instability at the far field, as confirmed by other researchers [18].

Hence, the focus of this study is to utilize the electrospinning setup with AVEs for producing CANFs mats and functionalized SWCNTs (f-SWCNTs) doped CANFs mats. The SWCNTs will be functionalized to improve the interfacial adhesion with nanofibers. Lastly, the effect of CANFs and f-SWCNTs doped CANFs mats on the mechanical properties of glass fiber composites will be evaluated using tensile, flexural, and impact tests to characterize the interlaminar strengthening mechanism.

## 2. Materials and Methods

### 2.1. Materials

The polymer solution used in electrospinning was prepared by mixing Polyacrylonitrile powder (PAN: density 1.184 g/cm${}^{3}$ at 25 °C) with N, N-dimethylformamide (DMF: 99% purity) supplied by Sigma Aldrich, Kempton Park, South Africa. The solution was stirred for 22 h at 50 °C until it became homogeneous. The composites were fabricated using Bisphenol-A epoxy (AMPREG 21 Resin) with woven E-glass mats supplied by AMT Composites Pty. Ltd., Johannesburg, South Africa. The SWCNTs with 99% purity were used to dope CANFs for strengthening the interlaminar region. The SWCNTs and the required functionalization chemicals, such as Polyvinyl alcohol (PVA) and dimethyl sulfoxide (DMSO), were purchased from Sigma Aldrich, Kempton Park, South Africa. The physical and mechanical properties of Bisphenol-A epoxy, woven E-glass, and SWCNTs are given in Table 1.

### 2.2. Testing Procedure

**Figure 1:**
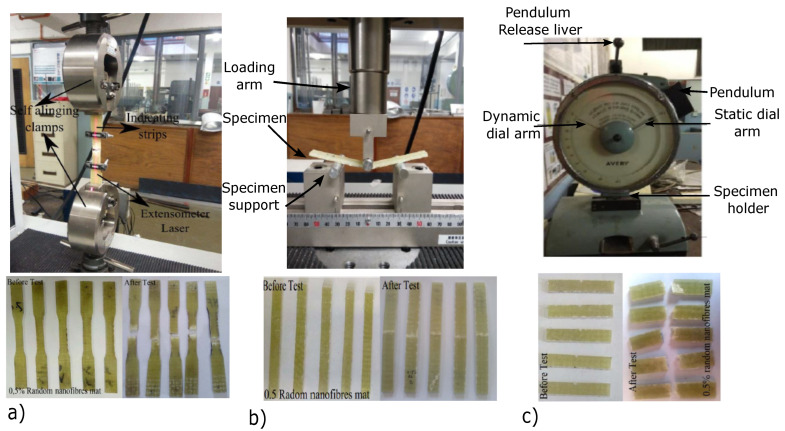
(**a**) Tensile testing setup and specimens. (**b**) Flexural testing setup and specimens. (**c**) Impact testing setup and specimens.

The tensile, three-point flexural, and Charpy impact tests were carried out on the composite specimens as per ASTM D638-08: 2010, ASTM D256-06a: 2010, and ASTM D790-07: 2010, respectively. The specimen thickness was kept at 3.2 mm. The flexural test specimens were fabricated with the thickness to width ratio of 1:16, and the gauge length between the supports was kept at 63 mm. A 20 kN SHIMADZU tensile testing machine was used to conduct both tensile and flexural tests (Figure 1a,b, which were carried out at a cross-head speed of 2 mm/min. The Charpy impact tests were carried out on a Tinius Olsen impact pendulum as per the ISO 179-1 standard (Figure 1c. The breaking energy was used to determine the impact resistance of the composite specimens.

### 2.3. Morphological Characterization

Morphological characterization of nanofibers and hybrid composite specimens was carried out using scanning electron microscopy (SEM-Nova 600 Nanolab and Quanta 400 FEG from FEI Company, Hillsboro, OR, USA) at the voltage range of 5 KV to 30 KV. Before analysis, the specimens were coated with a thin layer of carbon and a 15 nm layer of gold–palladium (Au/Pd) in a ratio of (60:40) in an EMITECH K950X evaporator and EMITECH K550X sputter coater, respectively. The specimens were then attached to 1 cm diameter stubs with DAG 580 colloidal graphite. The FTIR analysis was conducted on a Bruker Vector 22 FTIR spectrometer using 64 sample scans and 32 background scans. The Raman analysis was conducted using a Horiba Jobin-Yvon Lab RAM HR Raman spectrometer with a 514.5 nm line of an argon-ion laser as the excitation source.

### 2.4. SWCNTs Functionalization

The SWCNTs were functionalized using the Friedel–Crafts alkylation (FCA) process. The FCA process was carried out by mixing SWCNTs (0.4 g) and PVA (4 g) with a 40 mL DMSO solution in a 250 mL flask using a magnetic stirrer. The flask was then immersed in an oil bath for gradual heating to 90 °C in the presence of nitrogen. As the heating promotes the chemical process, a catalyst of aluminum chloride (ALCl${}_{3}$) was added for grafting the PVA chains onto the surface of the SWCNTs. During the chemical process, the OH groups from PVA reacted with ALCl${}_{3}$ and produced positive carbocations, (CH3${}^{+}$ ions: electrophiles), and negative ALCl${}_{2}^{-}$ ions, respectively. The electrophiles were then attracted to the SWCNTs’ hexagonal ring due to the CNT’s surface delocalized electron (nucleophilic) sites, and thus attached the PVA chains onto the SWCNTs. After 20 h, the chemical reaction was aborted by adding 100 mL of methanol/hydrochloric acid mixture (volumetric ratio 1:1). The solution was then centrifuged at 3500 rpm for 15 min to precipitate the functionalized SWCNTs (f- SWCNTs). The f-SWCNTs were washed, filtered, and dried in a furnace for 3 h at $70^{\circ }$.

**Figure 2:**
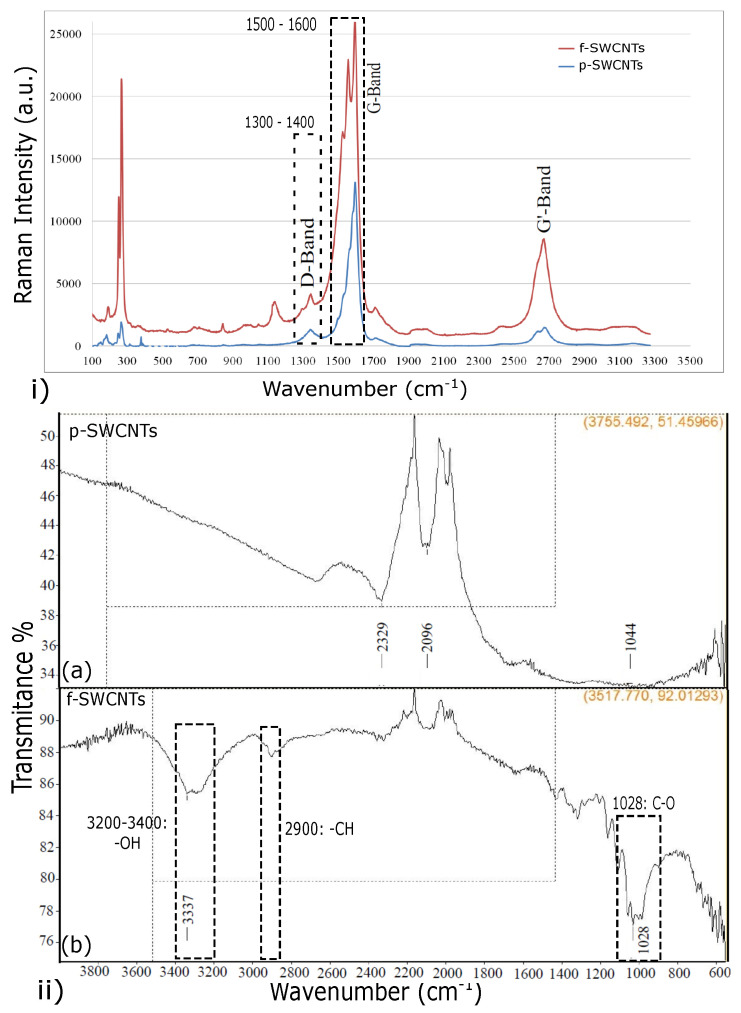
(**i**) Raman spectra of p-SWCNTs and f-SWCNTs. (**ii**) FTIR spectra of (**a**) p-SWCNTs and (**b**) f-SWCNTs.

The Raman spectra were used to analyze the functionalization results of both pristine (p-SWCNTs) and functionalized SWCNTs (f-SWCNTs) and are shown in Figure 2i. The two peaks at the wavelengths of 1300–1400 cm${}^{-1}$ and 1500–1600 cm${}^{-1}$ were for the disorder (D band) and graphite band (G band), respectively. These bands represent two different vibrational modes of SWCNTs. The D band represents the crystal disorder, such as sidewall defects (pentagons or heptagons) or Sp3 carbon hybridization, and the G band was for the Sp2 carbon hybridization. The ratio of D band to G band ($I_{D}/I_{G}$) increases with the increasing disorder [19] and defines the structural changes of the SWCNTs wall due to functionalization [20]. In the current work, the $I_{D}/I_{G}$ ratio was measured at 0.1025 and 0.1674 for p-SWCNTs and f-SWCNTs, respectively. The higher ratio of 0.1674 for f-SWCNTs was due to the carbocations attachment to the SWCNTs walls and the conversion of Sp2 to Sp3 hybridized carbons. This confirmed the PVA grafting to the SWCNTs wall during the FCA process [21].

Figure 2ii shows the FTIR spectrum for the range of 4000 to 500 cm${}^{-1}$ of both p-SWCNTs and f-SWCNTs. By analyzing the f-SWCNTs spectrum, the peak at 1028 cm${}^{-1}$ showed the C–O stretching, while the peak from 1100 to 1600 cm${}^{-1}$ confirmed the aromatic structures. The peaks corresponding to –CH and –OH bonds were obtained at 2900 cm${}^{-1}$ and 3200–3400 cm${}^{-1}$, respectively. These peaks confirm that the PVA chains are attached to the surface of SWCNTs.

### 2.5. Electrospinning of Aligned and Doped Nanofibers Mats

The electrospinning setup considered for this research consists of a DC voltage supply, NE-1600 syringe pump, and a grounded rotating drum collector (diameter 120 mm and width 210 mm), respectively. The electrospun nanomats were collected over the glass fiber mats attached to the rotating drum collector. The details of the complete setup with the spinning parameters were presented in our published manuscript [22] and the readers are encouraged to refer to the paper for further details. Following our previous experimental results, three electrospinning parameters, i.e., applied voltage, the distance between electrodes, and PAN concentration, were investigated in this work. In addition, auxiliary vertical electrodes (AVEs) were placed between the spinneret and collector. The range of tested and the optimized values of the spinning parameters are given in Table 2, which produced the nanofiber mats with fiber diameters in the range of 120–150 nm.

**Figure 3:**
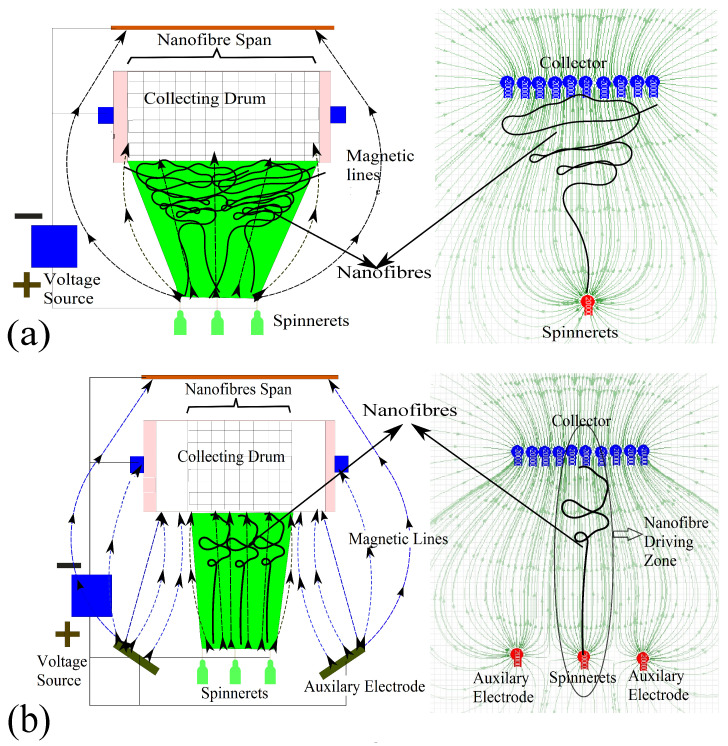
Electric field simulation software analysis (**a**) without AVEs; (**b**) with AVEs.

**Figure 4:**
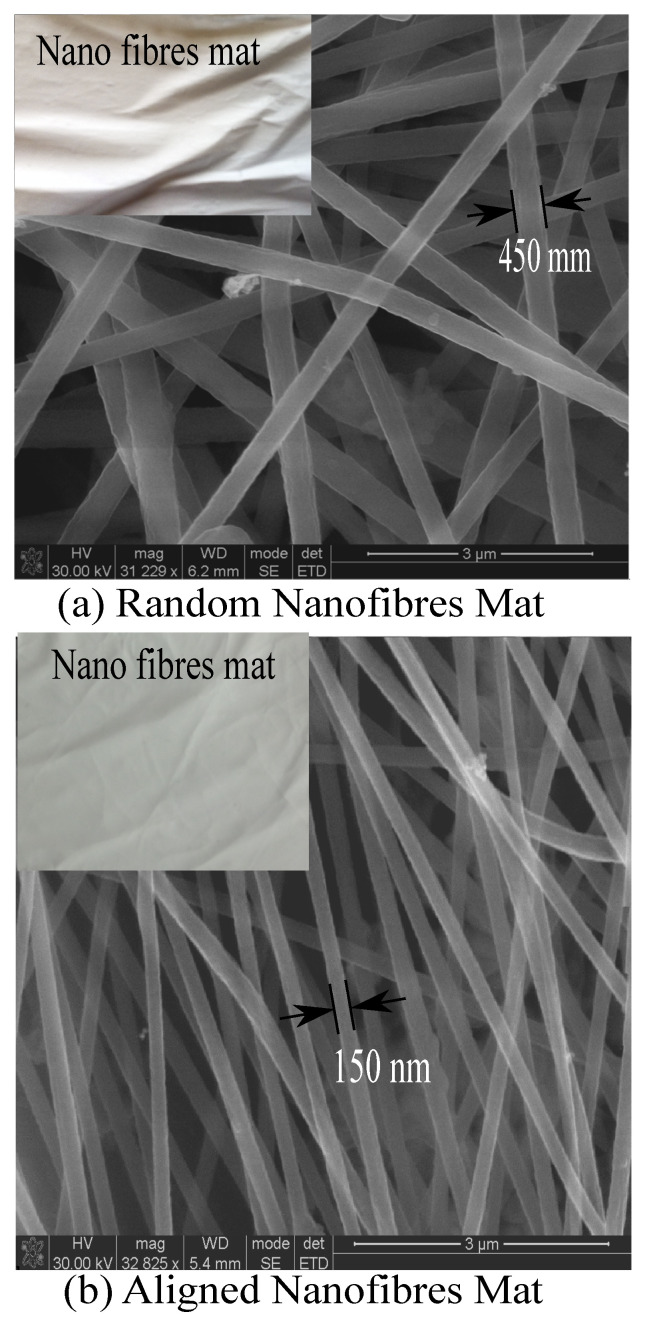
Electrospun nanofibers mats (**a**) without AVEs; (**b**) with AVEs.

To analyze the effect of AVEs on the whipping effect, an electric field simulation software was used to simulate the electrostatic forces. The software developed the electrostatic forces as electric field lines (green lines) between the spinneret and the collector. The positive electrode (the spinneret) is red, while the blue color refers to the negative electrode. The simulation shows that without AVEs, the electric field lines are spread over the entire width of the collector. As the electric fields were not focused on the collector, the whipping effect deposited the nanofibers randomly over the whole width of the collector (Figure 3a). However, after introducing AVEs (Figure 3b), the simulation shows that the electric field lines were narrowed down towards the center of the collector (nanofiber driving zone) by the AVEs electric field. This phenomenon reduced the whipping effect and aligned the nanofibers perpendicular to the collector axis. Figure 4 shows the camera images and scanning electron microscope (SEM) images of electrospun nanofibers mats produced without (Figure 4a) and with (Figure 4b) AVEs. As it can be seen, the AVEs significantly improved the alignment of nanofibers and further reduced the diameter of the nanofibers to 150 nm, which is 1/3 smaller than the nanofibers produced without AVEs. This confirms that the addition of AVEs significantly refined the nanofiber morphologies.

The electrospinning setup was used to produce the required nanomats. At first, the randomly oriented PAN nanofiber (RONFs) mats were produced without AVEs for the weight fractions of 0.1%, 0.2%, 0.5%, and 1%, respectively. Based on the experimental results, the weight fraction of 0.5 wt% PAN nanofibers were selected to manufacture continuous-aligned nanofibers (CANFs) mats using AVEs. Finally, the 0.5 wt% CANFs mats were doped with 0.25 wt% f-SWCNTs to produce f-SWCNTs doped CANFs mats. For doping, only 0.25 wt% f-SWCNTs were selected to minimize the density effect following the results from Pilehrood et al. [23]. Before spinning the nanomats, the required weight fractions of PAN-DMF solution with and without f-SWCNTs were prepared and electrospun over the glass fiber mats.

### 2.6. Composites Manufacturing Process

The composites were fabricated using a vacuum-assisted resin transfer molding (VARTM) method. Following our previous work [4], 32 vol% glass fiber composites were selected as a reference for producing nanomats strengthened hybrid composites. Considering the manuscript length, the details of the glass fiber composites experimental results are not included, and the mechanical properties for the 32 vol% glass fiber composites are given in Table 3.

**Figure 5:**
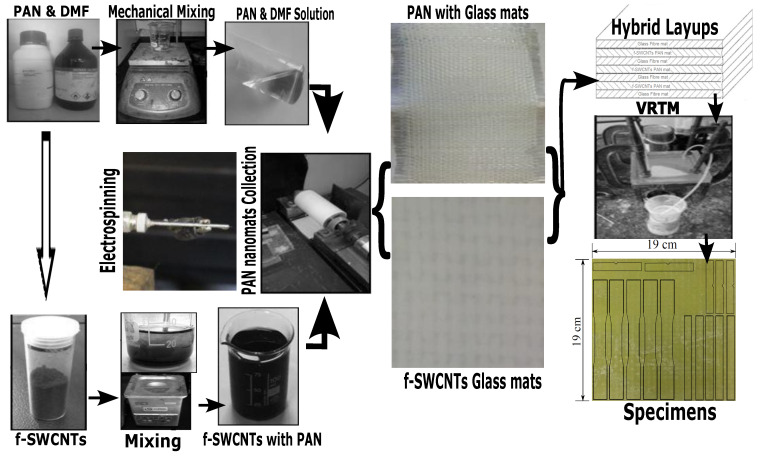
Fabrication process of glass fiber hybrid composites preform.

The VARTM mold consists of a female square steel ring, and upper and lower male plates, and these were assembled with the alternative layers of woven E-glass fiber mats coated with the RONFs, CANFs, and f-SWCNTs doped CANFs mats. Then, the resin-hardener mixer was placed at the mold suction port and the vacuum pressure of 80 psi was maintained until the resin filled the mold. Finally, the resin was allowed to cure for 24 h at room temperature. Figure 5 shows the manufacturing process of nanomats strengthened hybrid composites specimens, which were machined into tensile, flexural, and impact test specimens.

## 3. Results and Discussion

The discussions of the results are divided into two sections. The first section will focus on analyzing the results from RONFs mats strengthened hybrid composite specimens, and the CANFs mats and f-SWCNTs doped CANFs mats strengthened hybrid composites will be discussed in the second section.

### 3.1. RONFs Mats Strengthened Hybrid Composites

**Figure 6:**
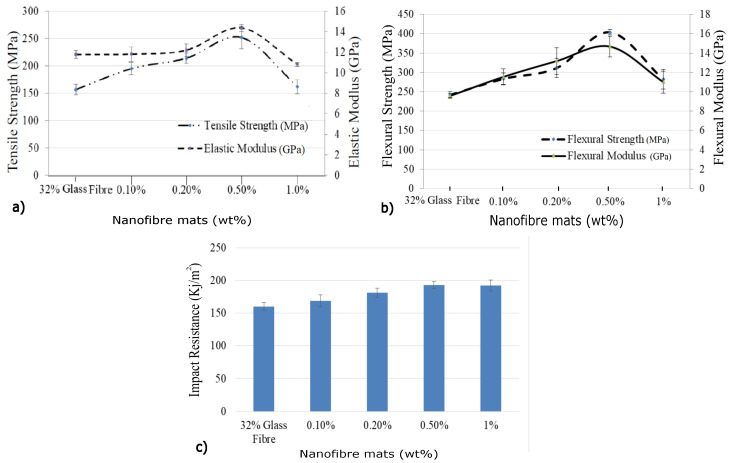
Properties of RONFs mats strengthened hybrid composite: (**a**) tensile properties; (**b**) flexural properties; (**c**) impact properties.

Figure 6 shows the mechanical properties of RONFs mats strengthened hybrid composites, and it uses the 32 vol% glass fiber composites as reference. The initial addition of 0.1 wt% of mats showed a scanty increase in properties compared to the 32 vol% glass fiber composites. Further addition of 0.5 wt% RONF mats showed an increase in tensile strength from 156.52 to 251.51 MPa, elastic modulus from 11.77 to 14.36 GPa, flexural strength from 242.2 to 404.01 MPa, flexural modulus from 9.58 GPa to 14.58 GPa, and impact resistance from 160.18 KJ/m${}^{2}$ to 193.112 KJ/m${}^{2}$, respectively. The statistical parameters for the RONFs strengthened glass hybrid composites are presented in Table 4.

**Figure 7:**
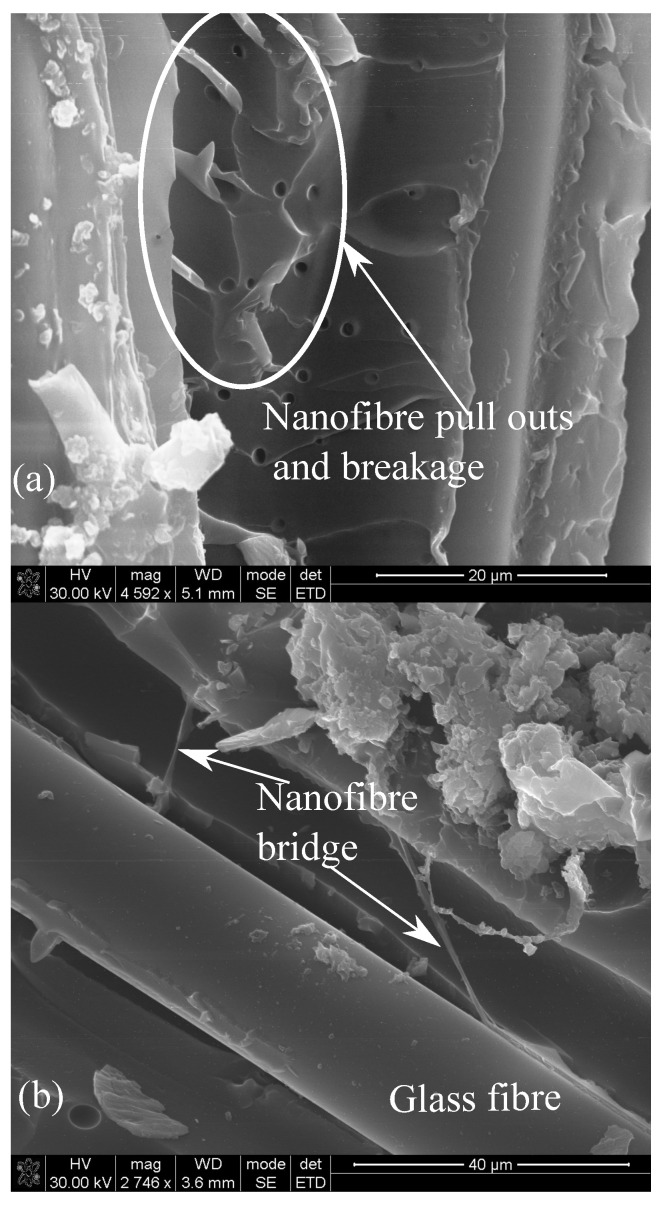
Fracture behavior of 0.5 wt% RONFs mats strengthened hybrid composite (**a**) Pulled out nanofibres (**b**) Bridging of nanofibres.

The increase in the properties could be attributed to the reinforcing effect of RONFs mats due to their higher specific surface area, which could have increased the contact area within interlaminar regions. The infused RONFs mats provided more resistance to the relative slippage of the matrix over the glass fiber mats while transferring the applied load (refer to Figure 7a) and thus improved the mechanical properties. In addition, the geometrical web network of RONFs mats provided bridging between the glass fibers and the matrix, as shown in Figure 7b, and thus increased the resistance to the applied load and improved the properties.

**Figure 8:**
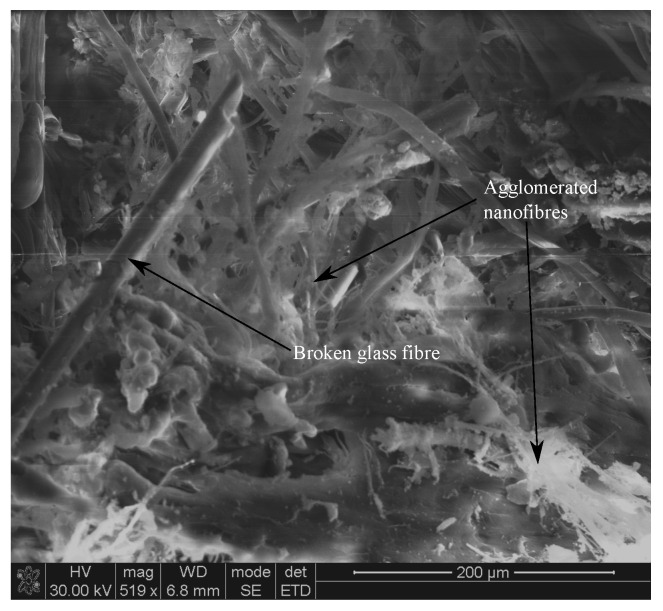
Agglomerated nanofibers of 1 wt% RONFs mats strengthened hybrid composite.

However, a similar effect was not observed for further increase in RONFs mats from 0.5 wt% to 1 wt%. This may be associated with the nanofibers agglomeration and poor penetration of resin into the nanofibers network due to the random orientation with higher weight fractions, which could have acted as stress concentration sites. Figure 8 provides evidence of agglomerated nanofibers within the interlaminar regions.

### 3.2. CANFs and f-SWCNTs Doped CANFs Mats Strengthened Hybrid Composites

Based on the above results, 0.5 wt% of nanomats were selected to manufacture 0.5 wt% CANFs mats and 0.25 wt% f-SWCNTs doped with 0.5 wt% CANFs mats strengthened glass fiber hybrid composites specimens to understand the effect of alignment and f-SWCNTs doping within the interlaminar regions. The experimental results used 0.5 wt% of RONF mats strengthened hybrid composites as a reference, and the results are presented below.

#### Mechanical Properties

**Figure 9:**
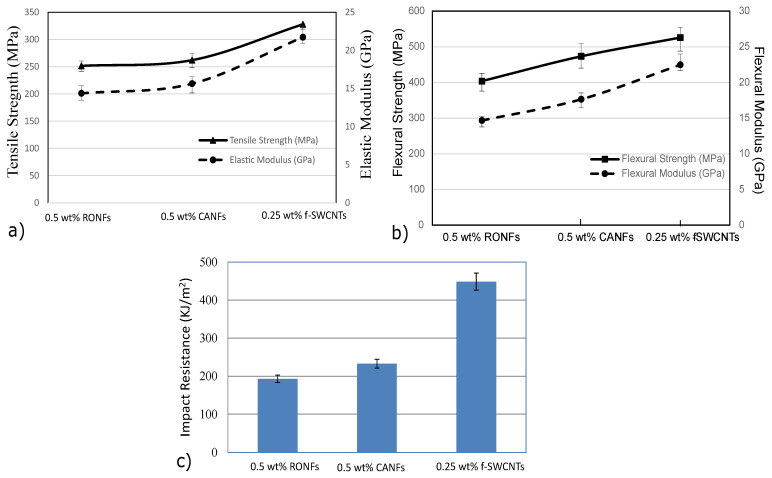
Mechanical properties of 0.5 wt% CANFs and 0.25 wt% f-SWCNTs doped with 0.5 wt% CANFs mats strengthened hybrid composites’: (**a**) tensile properties; (**b**) flexural properties; (**c**) impact properties.

The tensile, flexural, and impact properties of CANFs mats and f-SWCNTs doped CANFs mats strengthened hybrid composites are shown in Figure 9, and the properties values with percentage of increase are shown in Table 5.

The phenomenal increase in mechanical properties was possible due to the reinforcement effect of continuous/aligned nanofibers along with uniformly dispersed f-SWCNTs doped CANFs mats within the interlaminar regions. The increase in mechanical properties can be attributed to the molecular orientations, reduction in nanofibers diameter, and the uniform dispersion of the aligned nanofibers network due to AVEs. Further, the reduction in nanofibers diameters (450 nm to 150 nm) provided an approximately threefold increase in nanofibers network within the interlaminar region.

**Figure 10:**
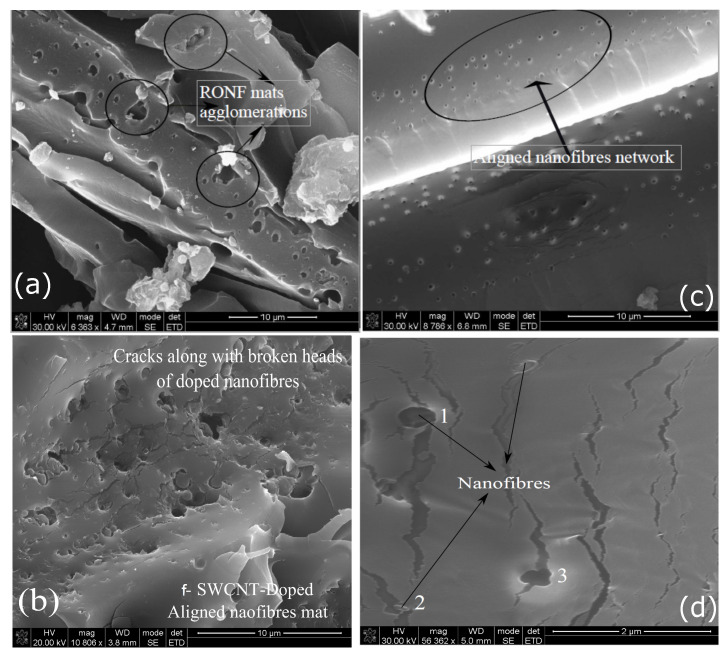
Fracture surfaces: (**a**) Agglomeration of RONFs mats strengthened hybrid composite tensile test specimen. (**b**) Tensile test specimen fracture surface of f-SWCNTs doped CANFs mats strengthened hybrid composite. (**c**) Fracture surface of the interlaminar regions with aligned nanomats networks of flexural test specimen. (**d**) Branched cracks within the interlaminar region due to nanofibers network.

Figure 10 shows the fractured surfaces of RONFs, CANFs, and f-SWCNTs doped CANFs mats strengthened hybrid composites. These images confirm that the RONFs mats (Figure 10a) accumulated nanofibers and created bigger voids as they were pulled out from the matrix. This led to an inefficient strengthening mechanism due to which the initiated cracks propagated without much resistance and resulted in poor mechanical properties. On the other hand, the CANFs mats with reduced diameter and improved distribution and orientation showed an orderly pull out of nanofibers and broken heads of nanofibers within the matrix (Figure 10c). This indicates that the bridging mechanism created by the nanomats networks acted as barriers for the propagating cracks, and, as such, the interlaminar regions were strengthened by the CANFs mats.

Figure 10b shows the SEM images of the fractured surface of f-SWCNTs doped CANFs mats strengthened hybrid composite. The images show several propagating cracks around the f-SWCNTs doped nanofibers heads. This implies that the initiated cracks were arrested by the f-SWCNTs doped CANFs mats and resisted/diverted the propagating cracks. The resistance prevented the separation of the matrix due to the propagating cracks. This phenomenon is evident in Figure 10d, where the cracks were either stopped or diverted due to the presence of f-SWCNTs doped nanofibers (refer to sites 1, 2, and 3). At site 3, the crack traveled across the nanofiber after the f-SWCNTs doped nanofiber was pulled out, and in sites 1 and 2, the cracks propagated over the broken nanofiber heads or pulled out f-SWCNTs doped nanofibers. This confirmed that the increase in mechanical properties of hybrid composites is due to the resistive f-SWCNTs doped nanofibers mats network within the interlaminar regions.

**Figure 11:**
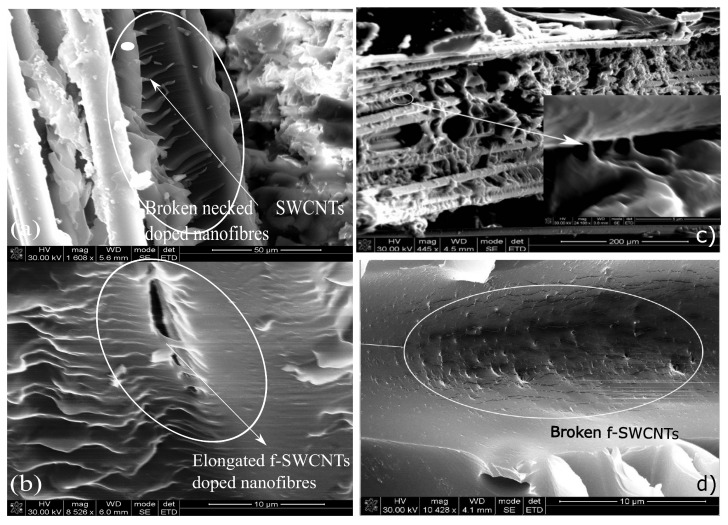
Fracture surfaces of 0.5 wt% nanofibers strengthened hybrid composites (**a**) Elongated f-SWCNTs doped CANFs mats in bridging. (**b**) Bridging of f-SWCNTs doped nanofibers. (**c**) Fractured surfaces of impact tested specimen. (**d**) Pulled and broken head of f-SWCNTs.

As discussed before, the bridging mechanism within the multiscale hybrid composites can be seen from the SEM images, as shown in Figure 11a,b. The functionalization of SWCNTs provided improved the dispersion, and further strengthened the bonding between f-SWCNTs and nanofibers, which could have resulted in a stronger nanofibers network within the interlaminar regions. Further, the alignment provided the required bridging, which resisted the crack propagation and promoted load transfer within the interlaminar region. Thus, the f-SWCNTs doped aligned nanomats strengthened hybrid composite provided the highest mechanical properties.

## 4. Conclusions and Recommendations

The continuously aligned nanofiber mats with and without f-SWCNTs were produced using an electrospinning setup with AVEs, which were then used to reinforce the interlaminar regions of glass fiber composites. The effect of aligned nanomats on the mechanical properties of glass fiber composites was seen from the experimental results; as such, both CANFs and f-SWCNTs doped CANFs produced hybrid composites with improved properties by strengthening the interlaminar properties. The addition of AVEs with the electrospinning reduced the nanofiber diameter and improved their alignments within the interlaminar regions. The reduced diameter and alignment of nanofibers helped to improve the molecular orientation and further helped to distribute f-SWCNTs uniformly into the glass fiber hybrid composites. The functionalization of SWCNTs promoted better dispersion of SWCNTs and improved the bonding between f-SWCNTs and nanofibers. These factors promoted better load transfer within the interlaminar regions and thus improved the mechanical properties of the glass fiber composites. The research could serve in different applications, such as electronics, filtration, etc., where aligned nanofibers with reduced diameter and improved molecular orientation are the essential requirements. The application of SWCNTs could also be extended to other research areas, such as smart textiles, transistors, information storage devices, batteries, EMI shielding, etc.

## Figures and Tables

**Table 1:** Properties of materials used—data collected from the suppliers.

Properties	Bisphenol-A Epoxy	Woven E-Glass Mats	SWCNTs
Tensile strength (MPa)	72.7–81.3	148.6–152.5	80,000–105,000
Tensile modulus (GPa)	3.3–4.3	7.2–8.3	∼1000
Elongation at break (%)	1.12	2.4	-
Density at 23 °C (gm/cm${}^{3}$)	1.148	2.6	1.7
Average diameter (nm)	-	-	0.83–1.3

**Table 2:** Spinning parameter values with AVEs.

Electrospinning Parameters	Range of Tested Values	Optimized Values
Concentration (%)	8–8.8	8.1
Flow rate (mL/h)	0.12–0.50	0.35
Applied voltage (DC-KV)	0–30	20
Collector to spinneret distance (cm)	5–35	25

**Table 3:** Properties of 32 vol% glass fiber composites.

	Tensile Strength (MPa)	Elastic Modulus (GPa)	Flexural Strength (MPa)	Flexural Modulus (GPa)	Impact Resistance (KJ/m${}^{2}$)
32 vol% glass fiber	156.52	11.77	242.2	9.58	160.18

**Table 4:** Statistical parameters of the RONFs strengthened glass fiber composites.

	Tensile Strength (MPa)				Elastic Modulus (GPa)				Flexural Strength (MPa)				Flexural Modulus (GPa)				Impact Resistance (KJ/m${}^{2}$)			
RONFs wt%	0.1	0.2	0.5	1	0.1	0.2	0.5	1	0.1	0.2	0.5	1	0.1	0.2	0.5	1	0.1	0.2	0.5	1
No of samples	5	5	5	5	5	5	5	5	5	5	5	5	5	5	5	5	5	5	5	5
Mean	194.47	213.56	251.51	161.58	11.79	12.09	14.36	10.79	283.77	311.70	404.01	282.62	11.55	13.18	14.58	11.01	168.82	181.40	193.11	192.02
Median	207.78	213.66	262.38	164.32	11.42	12.32	14.54	11.09	285.54	329.91	409.08	239.87	10.89	13.91	14.18	10.88	162.67	177.05	189.35	193.45

**Table 5:** RONF mats, CANF mats, and f-SWCNTS doped CANF mats hybrid composites properties and their increase.

Properties	0.5 wt% RONF Mats	0.5 wt% CANF Mats	% Increase	0.25wt% Doped with 0.5 wt% CANF Mats	% Increase
Tensile strength (MPa)	251.51	262.01	4.71	327.83	30.34
Elastic modulus (GPa)	14.36	15.63	8.84	27.72	93.04
Flexural strength (MPa)	404.01	473.44	17.19	525.96	30.18
Flexural modulus (GPa)	14.58	17.65	21.06	22.49	54.25
Impact resistance KJ/m${}^{2}$	193.11	232.75	20.53	448.58	132.29

## Data Availability

Data are contained within the article.
